# A Novel Call to Fix Medical Education: Pragmatic Steps to Encourage Dialogue and Advocacy for Providers and Medical Students

**DOI:** 10.7759/cureus.6606

**Published:** 2020-01-08

**Authors:** Christopher Gaeta, Joseph Cesarine

**Affiliations:** 1 Emergency Medicine, Children's Hospital of Philadelphia, Philadelphia, USA; 2 Emergency Medicine, Virtua Health, Berlin, USA

**Keywords:** medical eduation, education, gme, undergraduate education, liberal arts, nejm, humanities

## Abstract

Medical education in both undergraduate and graduate institutions has remained largely unchanged since the 1970s. Indeed, the demographics of providers have diversified accordingly to that of society's shifting sociocultural perspectives, this heightened transformation to represent women and minority populations have contrastingly not gained similar traction in medical education as the need for incorporating student creativity and humanities coursework in the training process of medical school. To adequately address the stagnant acceptance of liberal arts and humanities coursework for future physicians, it is critical to begin with restructuring the larger framework of medical education. More specifically, by increasing student participation in reflective and administrative discussions, and allowing trainees to be encouraged rather than hindered from creative modalities of their training. These initial systemic changes will provide the needed environment to allow providers to honestly discuss not only the humanities as a means of enriching physician as healers prating the art of medicine, but just as important, transforming the community to accept creative ideologies fosters a more refined means of supporting the future healers in our communities.

## Editorial

Not much has changed since the call for transforming undergraduate medical education some 40 years ago by Lewis Thomas’ publication in the New England Journal of Medicine (NEJM) [1]. This past year, physician Richard Ratzan offered a piece reflecting on the progress since Thomas' original NEJM article that began the dialogue discussing the importance of liberal arts education as a means of shaping our future physicians. Specifically, Ratzan's recent reflection about how medical schools have taken to these recommendations over the last 40 years concluded that “by all appearances, Thomas’ proposal did not go far, though the humanities have advanced undeniably in some curricula and campuses of undergraduate medical education.” [2] Indeed, Ratzan’s piece highlights this unfortunate reality that hardly any measurable progress has been made to lift up humanities majors in the medical school admissions process. Nonetheless, why not propose a pragmatic set of potential solutions to the current environment by respectfully shifting the discussion that Ratzan has focused heavily on attempting to provide validity to, instead why not excavate the complex contributing factors to this stagnant landscape across our medical schools that seems to continue to embrace the traditional lack of emphasis on humanitarian curricula in the undergraduate educational setting?

As a current student equipped with a unique insight to the admissions process from the applicant perspective as well as garnering a breadth of insight and pragmatic experience with the admissions side of the medical school process, it is clear that the lack of discussion to at the very least have a dialogue pertaining to this subject, augmented by a seemingly insignificant need to reform these changes from the medical school’s perspective has unequivocally fueled a lack of reform rather than discussion.

To preface this piece, I will not seek to root the subsequent content in a tangential justification in defense of the humanities as a means of educating our future providers. The very facts noted ad nauseam by Dr. Ratzan in his recent piece aptly defend the nearly half-century old documented publications pertaining to these claims. As such, I begin by exploring the objective truths relating to the current process for choosing and ultimately educating our future physicians. Quickly, it will become apparent that the concerning data warrants a call to unearth the deeper implications of the following practices.

Firstly, the complexities contributing to the lack of progressiveness in humanities within our undergraduate curriculum stems from a lack of change practically related to the curriculum of medical schools themselves. That being, it is not surprising that the shift from nearly all-white, homogenous medical school classes of men in the 1970s has progressed to the present day diversification in terms of race, gender, and ethnicity among the present Medical Doctorate (MD) and Doctor of Osteopathic Medicine (DO) degree programs. In fact, nearly half of the medical school classes in the nation as of this past year in 2018 are composed of almost a majority female class and almost a third of the entire graduates are minorities or Asian students. [2] However, why then has this transformation and call for inclusion not extended to the curriculum itself?

**Figure 1:**
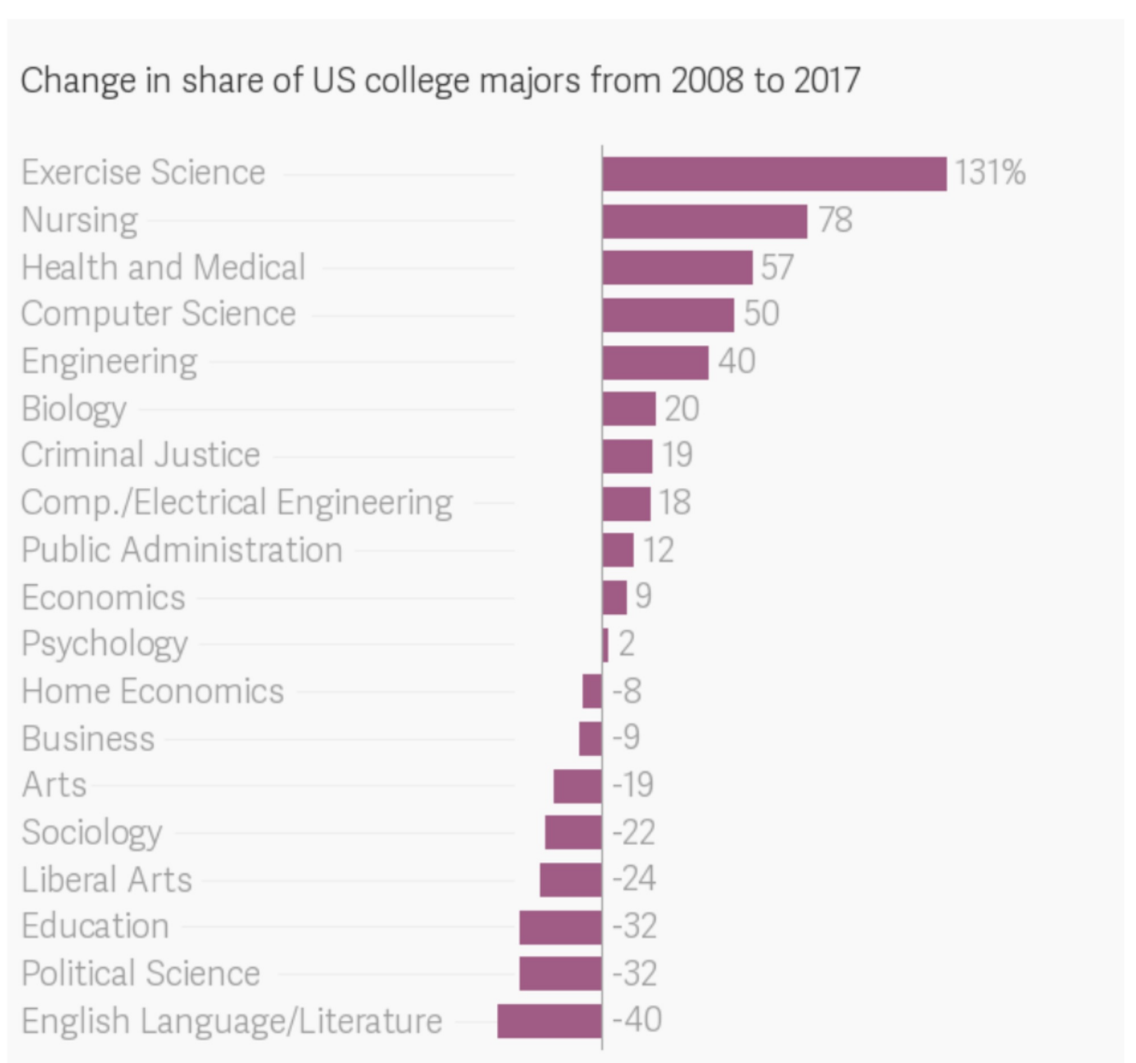
American Historical Association, Data from Atlas Survey Numerical values denote the percent change in degrees attained in the respective major category from 2008 to 2017. E.g. Nursing majors had a 78% increase in the number of degrees attained in the major from 2008 to 2017.

Many would point to the diminishing interest overall in the humanities as a college major. That is, perhaps the absence of humanities in medical education simply is the result of a decrease in the overall popularity for students as a whole to pursue this discipline? Specifically, the fact that nearly all of the college majors seeing negative growth in overall degree attainment from undergraduates over the most recent decade have been those in the humanities. Figure 1 depicts this negative percent change with a decrease close to 25% of overall graduates pursuing a liberal arts course of the study compared to stark growth in the sciences and similar STEM majors. [3] 

 

Nonetheless, it is not convincing from the available data that the sole impact of the lack of liberal arts integration directly correlates with the lack of critical mass, or in other words, lack of enough students voicing interest in studying these disciplines. Indeed, the diminishing appeal of striving to master humanitarian bodies of work correlates with a tendency for less individuals to seek these courses of study. However, this does not remain the leading reason why the "art" portion of medical practice is being left secondary to scientific skills set education in medical school despite the common phrase calling “Medicine as much an art as it is a science.” [1] 

What then is causing this lack of change in creativity and interpersonal focus of the medical school and premedical studies educational focus? The answer is as shocking as it is simple: how can we allow for our healers to pursue, or at least be encouraged to pursue an education in the liberal arts when we lack the professional support and enrichment to explore nonquantitative and nonmedically centered academic experiences if we fail to respect our trainees and encourage analytic problem solving pertaining to their own education rather than the one-dimensional didactic and nearly unwavering privatization of medical training refining the art of learning how to diagnose your patient. In turn, we have missed the unequivocally more vital opportunity in this educational experience: the societal, cultural, and dozens of other complexities attributing to your patient presenting to you. 

How can we expect authentic progress in medical care? It is clear to see why our care system has continued to foster this shortcoming in training future providers when students are not being encouraged to actively partake in reformation of educational modalities in addition to a total oversight that has been present for decades in which we are educating physicians to focus on the objective nuances of cases rather than subjective, societal implications, and contributions to your patients. 

Of the scarce data sources available, the Association of American Medical Colleges (AAMC) provides a particularly robust archive of nationwide data surrounding medical college admissions. The ultimate feedback from administrators and students offers a host of quantitative pearls. 

**Figure 2:**
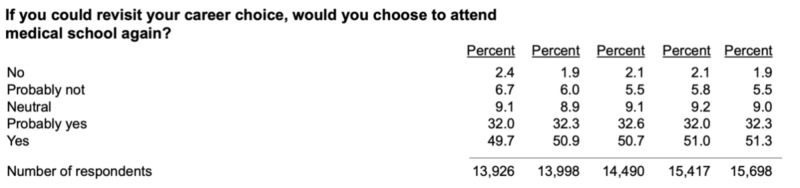
Association of American Medical Colleges (AAMC) Medical School Enrollment Survey, 2015-2019 Annual Data Surveying results from the AAMC conducted annually from 2015 to 2019. The respondents noted their answer to the bolded question by marking one of the five answer choices on the left-most column of the table. The vertical columns of results represent the percentage distribution of respective answers for each year surveyed. E.g. The distribution of responses for the year 2015 includes responses to the above question in which 2.4% of respondents answered no, 6.7% answered probably not, 9.1% answered neutral, etc.

For example, data in Figure 2 from the AAMC in 2019 concluded that 27.7% of recent medical school graduates do not agree or strongly agree that their MD or DO program did a good job fostering their development as a person [4]. Unfortunately, the results unravel even more cause for discussion. A total of 71.8% of recent medical school graduates do not agree or strongly agree that their MD or DO program’s dean of students or similar office on campus has adequately integrated student representation on key medical school committee and also 66.4% of recent medical school graduates do not agree or strongly agree that their MD or DO program’s dean of students or similar office on campus has provided an adequate responsiveness to student problems. [4] 

How then can we blame students for a lack of authentic, interpersonal development in their training when a majority of them feel that their institutions are neither responsive to their concerns nor partake in decision-making processes that would provide the opportunity to share their thoughts on reforming the undergraduate education system. If you went to intern for a company for nearly half a decade and were not encouraged to impart your thoughts on the experience or ways to improve the experience, how can we expect change with these processes. The same parallel should be equated to undergraduate medical education lacking the progression that Dr. Thomas hoped to see take form in the 1970s. 

How then, can we authentically make a call to embrace the Art of Medicine truly being incorporated into education going forward? Firstly, I challenge providers and medical school administrators to attempt to begin to encourage students and recent graduates to join the conversation. We see a potential benefit (at the very least) to encourage humanities majors to represent an increased portion of the MD and DO population, but we need to begin by encouraging creativity and authentic dialogue amongst one another because this is the path forward to slowly enact changes in the medical education system.

All of this just noted has omitted the equally pressing concerns that plague our future medical doctors from ever-present burnout kindled by an increasing shortage of physicians in the United States. This challenge is further augmented by increasing student debt levels of medical students and a lack of training sites for our students for residency and fellowship programs. 

With this doubt, you may ask an optimistic student seeking to advance along the path of medical training: why would you be incentivized to go into a career in which we are plagued by this doubt affirmed by nearly half of doctors wishing that they did not go into this field if given the chance to switch their professions. As such, it is very challenging for a potential medical student to look past these harsh realities to authentically unearth the deeper truths of the duties of physicians. I hope to one day reinstill the remarkably humbling benefits offered to medical doctors who have the chance to practice the most noble possible career that commits your very being to heal our fellow man. [5]

As such, why not advocate for a transformation in our medical institutions and professionals alike? In particular, encouraging advocacy and feedback within the medical community is a first step toward shifting the paradigm away from the shortcomings associated with the duties of a medical doctor. We can begin to support humanities as a means of educating the creative side of our providers only when the systems and institutions that govern educational programs for medical doctors equip future providers to be an active advocate for their colleagues and creatively refining their capacity to reach within oneself and imagine the internal struggles and experiences faced by patients seeking treatment.
